# First record of the leafhopper *Platymetopiusnotatus* Fieber (Hemiptera, Cicadellidae, Deltocephalinae) from northwest Algeria with description of the species

**DOI:** 10.3897/BDJ.9.e71418

**Published:** 2021-09-03

**Authors:** Nabila Guerrouche, Kamel Hamadi, Faiza Marniche, Hocine Aziri

**Affiliations:** 1 Laboratory of Dynamics and Biodiversity, FSB, USTHB, LP 32 El Alia, Bab Ezzouar, Algiers, Algeria Laboratory of Dynamics and Biodiversity, FSB, USTHB, LP 32 El Alia, Bab Ezzouar Algiers Algeria; 2 National Institute of Higher Education for Youth Executives Tixeraine, Algiers, Algeria National Institute of Higher Education for Youth Executives Tixeraine Algiers Algeria; 3 Higher National Veterinary School El Harrach, Algiers, Algeria Higher National Veterinary School El Harrach Algiers Algeria; 4 Expertise and Consulting Company in fruit production techniques, Sarl Djezagri, Algiers, Algeria Expertise and Consulting Company in fruit production techniques, Sarl Djezagri Algiers Algeria

**Keywords:** Khemis Miliana, pear orchard, leafhoppers, morphology

## Abstract

**Background:**

A continuous monitoring of leafhoppers in a pear orchard (*Pyruscommunis* L.) of Santa Maria variety, located in Khemis Miliana in northwest Algeria for a period of 7 months from February to August 2019, revealed a species, *Platymetopiusnotatus* Fieber, earlier having been reported from Europe (Spain, Portugal). The species is reported in two African countries (Morocco and Tunisia).

**New information:**

The species is recorded for the first time from Algeria and is re-described and illustrated along with the male and female genitalia structures for the first time.

## Introduction

The leafhopper family Cicadellidae is the largest family amongst Hemiptera belonging to the suborder Auchenorrhyncha. It can be easily recognised by the rows of macrosetae on the hind tibiae and by a transverse suture dividing the mesepimeron amongst the families of Auchenorrhyncha (Dietrich 2005). The subfamily Deltocephalinae is the largest leafhopper subfamily and consists of 7270 valid species and 969 genera, placed in 39 tribes (Zahniser 2007), occurring in all zoogeographic regions (Zahniser and Dietrich 2008). Deltocephaline species are predominantly found on grasses, but they also occur on shrubs and trees (Dai et al. 2006), most of them feeding on phloem sap, using needle-like mouthparts. Due to their feeding habitat, many of these insects are vectors of economically important plant diseases (Brčak 1979), through either direct feeding or indirectly by transmission of plant pathogens, such as viruses and bacteria, like phytoplasmas and other mollicutes (Nielson 1979,Weintraub and Beanland 2006).

The genus *Platymetopius* Burmeister is considered difficult and in need of revision. Indeed, as already pointed out by Dlabola (1974) and Abdul-Nour (1987), it is a large and hetrogeneoues genus with several species inadequately described or known only from the female. At present, 77 valid species are known from this genus and widely distributed in the Palaearctic, Oriental and Afrotropical Regions (Zahniser 2007). In Algeria, two species are already recorded, *P.major* Kirschbaum and *P.apicalis* Puton. The latter species is probably endemic (Metcalf 1967, Nast 1972). From this study, we add *P.notatus* Fieber to increase the number to three species from Algeria.

## Materials and methods

**Figure 1.**
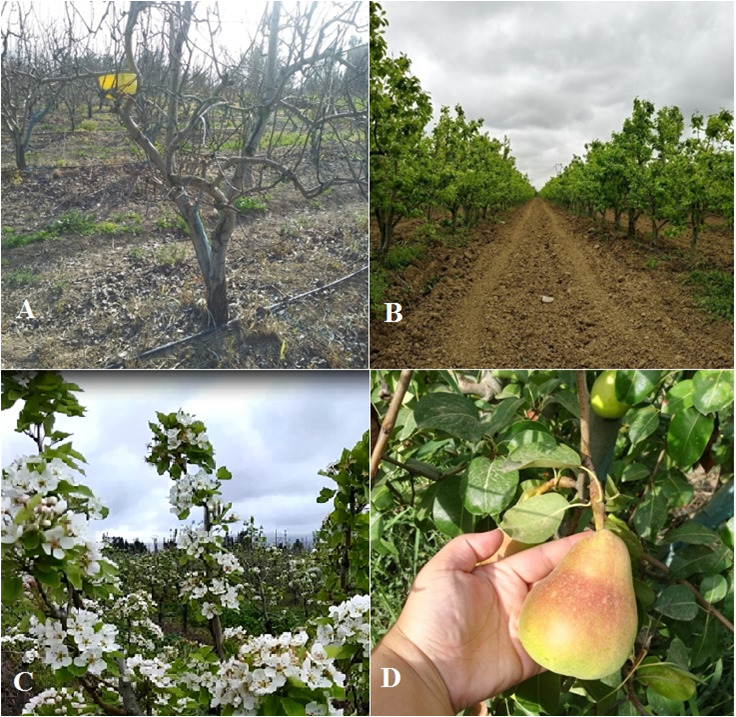
Pear tree orchard, Khemis Miliana (Photos by Guerrouche N., 2019), **A** yellow pan-trap, **B** general aspect of the pear orchard, **C** flowering period of the pear trees, **D** the pear fruit in the harvest period (middle August).

In Algeria, great undiscovered richness of entomofauna species still exist because of the different topographic conditions, the large diversity of vegetation and various kinds of climate. As part of doctoral research on insect pests associated with pear trees, insect samples were taken from February 2019 to August 2019, in an agro-ecosystem of pear orchards in Khemis Miliana City (Ain Defla). It is about 120 km away from Algiers. The plain of Khemis Miliana has an agricultural vocation, characterised by a semi-arid continental climate with dry summers and rainy winters. The surface area of the prospected parcel is 3 hectares containing 1200 trees planted in lines with a spacing of 1.50 m (Fig. 1). The prospected parcel has benefited from regular cultivation work. Weeds between the trees were removed and incorporated in soil between rows of pear plants in order to enrich the soil with organic matter. Its soil is of clay-silty type. For the phytosanitary monitoring of the orchard, the products used are precisely deficiency correctors, fungicides, acaricides and insecticides. The choice of the insecticides is based on the main insect pests (pear psyllid and codling moth) of the crop under study. The leafhoppers were trapped by setting up yellow pan-traps charged with salt solution (250 gm of sodium chloride (NaCl) dissolved in one litre of water with a few drops of odourless soap solution) and placed amongst the foliage of pear tree at a height of 1.50 m above ground level. The traps were placed from February to August 2019, but only in the period from June to August, we found the leafhoppers. The specimens collected were preserved in 70% ethanol in vials with airtight screw caps and labelled with date of collection, place and the number of yellow pan-traps. The specimens were examined under a stereoscopic microscope for detailed morphological characters. For studying the male genitalia characters, the abdomen was removed from the specimen which was dried and mounted on card with appropriate label data previously and macerated in hot 10% potassium hydroxide (KOH) till the soft tissues dissolved, the abdomen was then removed to distilled water, washed and then transferred to glycerol. We have followed the procedures advocated by Oman (1949) and Knight (1965) for the dissection of male and female genitalia which was done under a stereoscope microscope in glycerol and, after imaging male and female genitalia along with the remaining part of abdomen, these were stored in a microvial containing glycerol and stoppered with neoprene cork and pinned under the specimen from which the abdomen was removed. Habitus photos were taken using a Leica M205C stereo binocular microscope, equipped with a DFC 425 Camera. Images of male genitalia were taken using a Leica DM 2000 compound microscope, equipped with a camera Lucida and a DEC 425 camera. Partially focused images of each specimen or structure were stacked using the Zerene Stacker software.

The specimens used in this study are deposited in the laboratory of Dynamic and Biodiversity, University of Sciences and Technology Houari Boumediene, Algiers, Algeria and University of Agricultural Sciences, Bangalore, India, “UASB” collection.

## Data resources

There were 69 specimens of *Platymetopiusnotatus* Fieber amongst the leafhoppers trapped in the yellow pan-traps and formed the dominant group. Other leafhoppers collected were Aphrodinae (Aphrodini), Megophthalminae (Agalliini), Deltocephalinae (Athysanini, Deltocephalini, Eupelicini, Goniagnathini, Opsiini and Paralimnini) and Typhlocybinae (Empoascini) and these will be dealt with in other papers that will follow this. The leafhoppers from the genus *Platymetopius* were found only in the period from June to August. There was no apparent damage caused by *Platymetopius* on the foliage or on the plant growth of pear trees. The following works, Ribaut (1952) and Ossiannilsson (1983), were used to determine the genus *Platymetopius*. All the collected specimens were identified by Guerrouche N. and Prof. Marniche F. to the genus levels. A small sample of leafhoppers, collected during this study, were sent to Dr. C.A. Viraktamath for specific determination and confirmation. The species level identification of *Platymetopiusnotatus* was first made by Dr. C.A. Viraktamath using the original descriptions of species known from the northwest Africa as *P.notatus* which was later confirmed by Dr. I. Malenovsky. The species of *Platymetopius* involved is described and illustrated hereunder.

## Taxon treatments

### Platymetopius notatus

Fieber, 1869

354544A8-222B-5EE4-96AD-EDAA9300C6A7

Platymetopius notatus Fieber, 1869

#### Materials

- **Type status:**Other material. **Occurrence:** recordedBy: Nabila Guerrouche; individualCount: 69; sex: "male", "female"; **Taxon:** scientificNameID: *Platymetopiusnotatus*; parentNameUsage: Fieber, 1869; kingdom: Animalia; phylum: Arthropoda; class: Insecta; order: Hemiptera; family: Cicadellidae; genus: Platymetopius; specificEpithet: *notatus*; taxonRank: species; **Location:** locationID: Khemis Mliana; higherGeographyID: Algeria; higherGeography: North Africa; continent: Africa; country: Algeria; municipality: Ain Defla; minimumElevationInMeters: 110 m a.s.l.; maximumElevationInMeters: 250 m a.s.l.; verbatimCoordinates: "36.0026389N", "2.195691666E"; verbatimLatitude: "36.0026389N"; verbatimLongitude: 2.195691666E; decimalLatitude: 36.0026389; decimalLongitude: 2.195691666; **Identification:** identifiedBy: Chandra A Viraktamath; dateIdentified: 2021; **Event:** samplingProtocol: Yellow pan

#### Description

**Figure 2.**
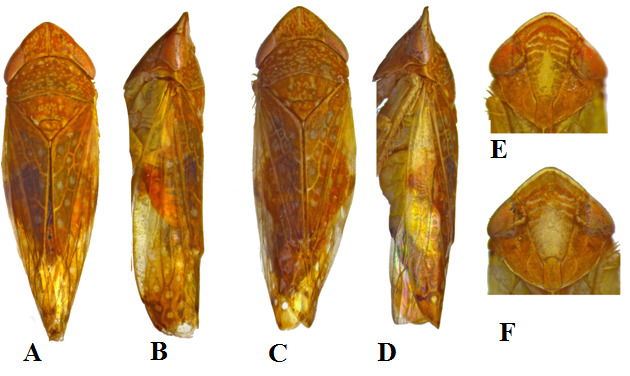
*Platymetopiusnotatus***A** habitus, dorsal view, male, **B** habitus, lateral view, male, **C** habitus, dorsal view, female, **D** habitus, lateral view, female, **E** face view, male, **F** face view, female.

**Male.** Reddish-orange with pale-yellow markings on head, thorax and forewings. Crown with median longitudinal stripe enclosing coronal suture and numerous spots (slightly longer than wide) on either side of this pale yellow median stripe. Face reddish-orange, with the following pale yellow: border along anterior margin, arcuate dashes on either side of the median line and a large median elongate oval area on frontoclypeus. Eyes reddish with median transverse pale yellow band along anterior margin. Pronotum and mesonotum reddish-orange, mottled with small round pale yellow spots; pronotal lateral and posterior margins narrowly pale yellow. Forewing reddish-orange with transparent small round spots on clavus and inner side of corium, outer half of corium with basal and sub-basal triangular hyaline areas, apical cells with a central hyaline round spot surrounded by pale brown area. Pleurites, sternites and legs yellowish (Fig. 2A-B and E).

**Female.** Similarly coloured as male, but the frontoclypeus largely pale yellow in the lower half (Fig. 2C-D and F).

**Structure**: Crown triangular, disc slightly concave, with transition from crown to front sharp, but not carinate, about 0.7 times as long medially as distance between eyes. Face about as long as wide including eyes, transclypeal suture well-developed. Pronotum as wide as head including eyes or slightly narrower, about as long medially as crown, lateral margins carinate, disc slightly convex in lateral view. Mesonotum longer than pronotum. Forewing exceeding abdomen in both sexes, venation reticulate in both clavus and corium and apical cells subdivided by accessory cross veins, appendix well developed.

**Male genitalia**: Pygofer without anterior apodemes, dorsum well sclerotised, about as long as its height in lateral view, dorsal margin before apex with a group of macrosetae; posteriorly narrowed with posterodorsal angle produced dorsally into a spine-like process exceeding dorsal margin. Subgenital plate more or less triangular with bluntly rounded apex, with thin uniseriate macrosetae located sub-marginally on ventral surface. Style with apophysis stout and slightly curved, with well-developed pre-apical lobe. Connective Y-shaped, arms shorter than stem, apex of stem bifid distally. Aedeagus with short dorsal apodeme, shaft directed posteriorly in basal third, then directed dorso-anteriorly, shaft with apical gonopore and with laterobasal pair of processes about as long as shaft, similarly curved as shaft and are slightly bent laterally near apex. Anal segments large, exceeding posterior apex of pygofer, without processes, segment X dorsally well-sclerotised (Fig. 4).

**Figure 3.**
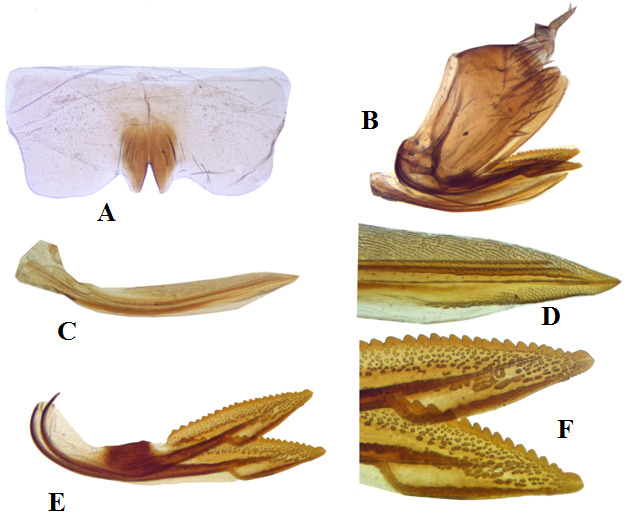
*Platymetopiusnotatus*, female **A** sternite VII, ventral view, **B** female genitalia, lateral view, **C-D** valvula I, lateral view, different magnifications (×20, ×40), **E-F** valvula II, lateral view, different magnifications (×20, ×40).

**Figure 4.**
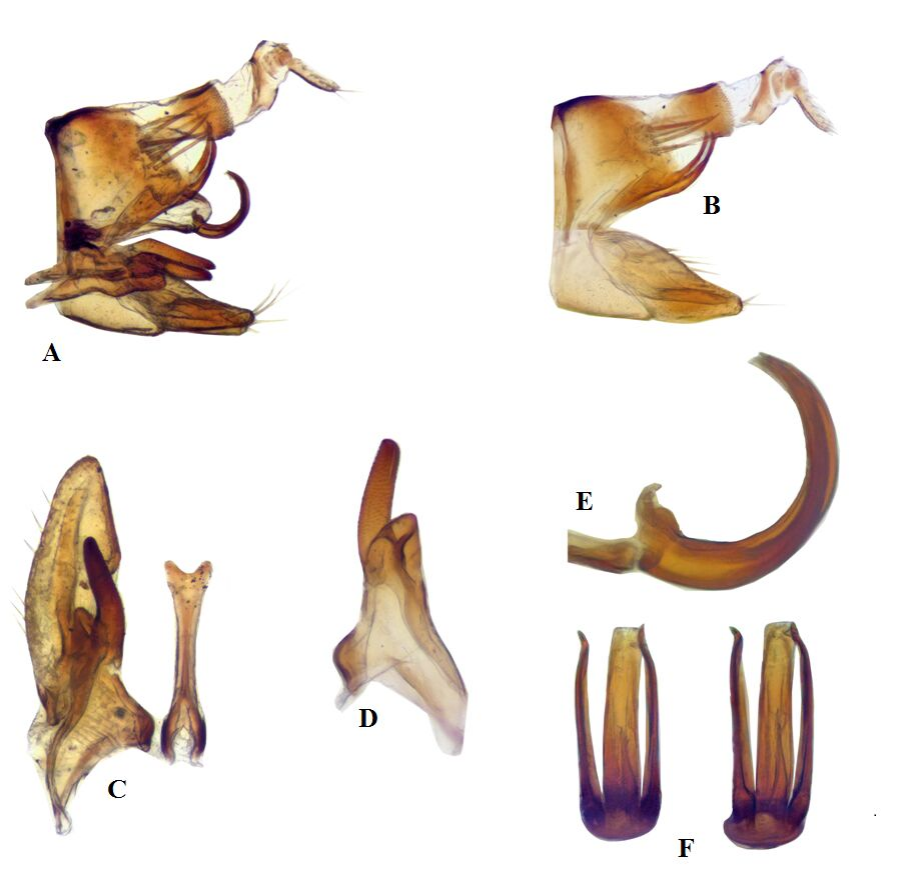
*Platymetopiusnotatus*, male genitalia **A** genital capsule, lateral view, **B** pygofer, valve and subgenital plates, lateral view, **C** subgenital plate, valve, style and connective, dorsal view, **D** style, dorsal view, **E** aedeagus and part of connective, lateral view, **F** aedeagal shaft and laterobasal processes, posterior view. Magnifications (×20, ×40).

**Female genitalia**: Sternite VII more or less transverse, twice as wide as long medially, posterior margin with median sclerotised lobe with median narrowly V-shaped incision. Valvulae I and II slightly curved. Dorsal sculpturing of valvula I strigate, reaching dorsal margin and occupying distal 0.60 length. Valvula II with denticular area slightly expanded, preceded by hyaline area and narrowed distally, occupying distal half, teeth prominent, with few secondary dentition (Fig. 3).

**Measurements**: Male 4.9 mm long, 1.5 mm wide across eyes. Female 5.5 mm long and 1.7 mm wide across eyes.

**Material examined**: 26 males and 43 females, ALGERIA: Khemis Miliana, 36°14’9.5’’N, 2°11’44.49’’E, June - August 2019, off pear tree, yellow pan-trap, Nabila Guerrouche coll.

**Figure 5.**
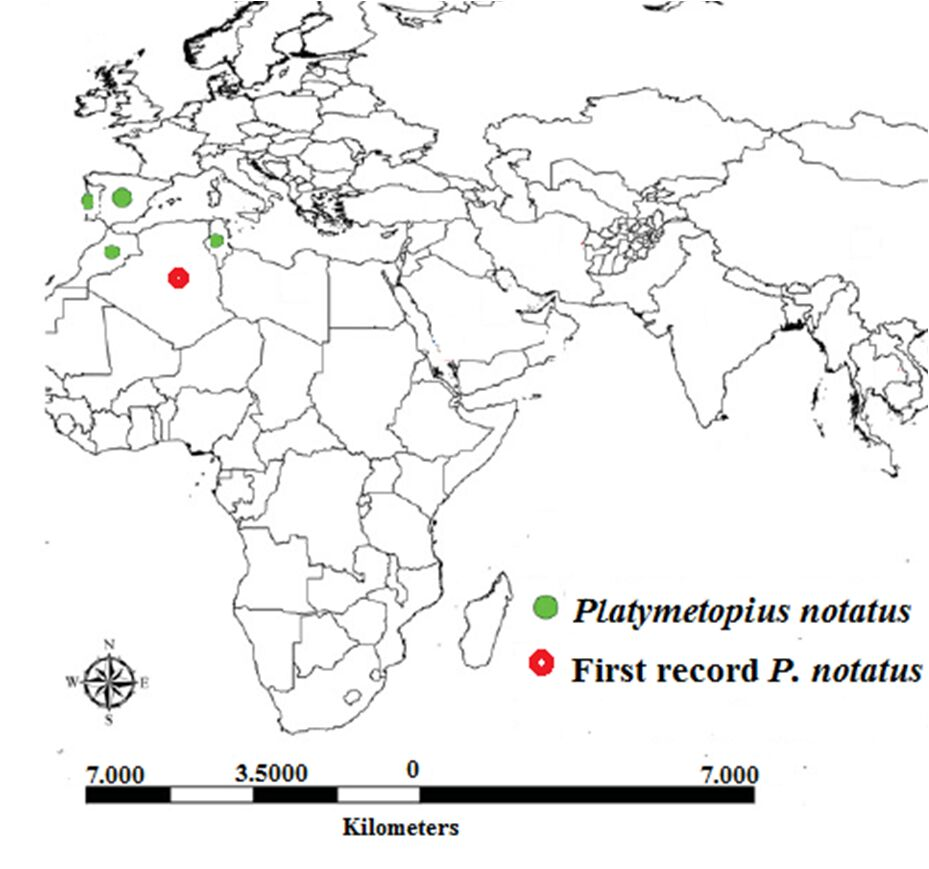
Map of the distribution of *Platymetopiusnotatus* at global scale.

**Distribution**: Morocco, Portugal, Spain (Metcalf 1967), Tunisia (Nast 1972), Algeria (new record) (Fig. 5).

## Discussion

The genus *Platymetopius* is one of the large genera of the tribe Athysanini of the subfamily Deltocephalinae with more than 77 known species from the Palaearctic, Oriental and Afrotropical Regions. Most leafhopper species can only be recognised, based on the structure of the male genitalia and, for many species including *P.notatus*, this information is not available leading to difficulty in its accurate identification. Nast (1972) listed five species of the genus from northwest Africa, namely *P.apicalis* Puton, *P.major* Kirsc hbaum, *P.notatus* Fieber, *P.perplexus* Linnavuori and *P.ziziphi* Bergevin, amongst these, only two, *P.apicalis* and *P.major*, were reported from Algeria. *P.major* is widespread in the Palaearctic Region (Metcalf 1967, Nast 1972). Dlabola (1981) removed the species *P.ziziphi* as the type species of his new genus *Masiripius* Dlabola. *P.major* differs from *P.notatus* in the structure of the pygofer process which is long and curved with subapical leaf-like expansions (short, simple and straight process in *P.notatus*), laterobasal processes of aedeagal shaft are longer than shaft and are strongly curved dorsoanteriorly and the aedeagal shaft is with lateral apical short processes (in *P.notatus*, the lateral basal processes are not strongly curved and are about as long as the shaft and the aedeagal shaft apex lacks the lateral short processes). Linnavuori (1965), in describing *P.perplexus*, did not illustrate the male genitalia, but said the genitalia are similar to that in *P.filigranus* Scott, which was figured by Ribaut (1952) and has much shorter pygofer process and longer and subapically expanded laterobasal processes of the aedeagal shaft which are also longer than the aedeagal shaft. The male genitalia of *P.apicalis* and *P.notatus* were not previously illustrated. *P.apicalis* was described from Algeria by Puton. In the original description, Puton (1877) states that the vertex is a little prolonged and not very acute, hardly longer than broad behind and between the eyes; there is a milky white line on its anterior edge on each side of the median line and up to the eyes; its surface is of a beautiful black, except for a transverse white band on its posterior edge which, nevertheless, only touched on its median line and a small punctiform white spot at the same angle of the top and sometimes, perfectly isolated, sometimes united to the anterior white line. Hence it is quite different from the species that has been collected from pear trees in this study. The male and female genitalia of the specimens, collected in this study, exactly match those of specimens collected from Morocco and determined by Dr. J. Dlabola and Prof. H. Ribaut (Dr. Igor Malenovsky, personal communication to Dr. C.A. Viraktamath, see Acknowledgements for details). In fact, Fieber (1969) describes *P.notatus* as follows: crown is short, marbled rusty yellow, a little longer than half, broad at the nape, with yellowish-white central stripe and margin. Pronotum is brown with small white spots, forehead yellow, above with rusty yellow angular lines and a few side lines. Cheeks and clypeus are brown yellow. Clavus is brown yellow, in corium, short from the middle a large brown triangle, from the final seam to the edge, brown yellow inside down to the first end cell. Female pregenital segment transversely wide, a small blunt tooth in the middle of the posterior margin and the sides blunt-cornered in the middle and with a brownish-yellow central stripe. The male and female genitalia of *P.notatus* are described and illustrated for the first time.

## Supplementary Material

XML Treatment for Platymetopius notatus

## References

[B7385964] Abdul-Nour H. (1987). Studies on the genus *Platymetopius* Burmeister, 1838 in the Near East, with description of seven new species (Homoptera: Auchenorrhyncha, Cicadellidae). Mitteilungen der Schweizerischen Entomologischen Gesellschaft.

[B7381666] Brčak J, Maramorosch K, Harris K F (1979). Leafhopper vectors and plant disease agents.

[B7297038] Dai W, Zhang Y, Viraktamath CA, Webb MD (2006). Two new Asian Scaphytopiini leafhoppers (Hemiptera: Cicadellidae: Deltocephalinae) with description of a new genus. Zootaxa.

[B7295572] Dietrich C. H. (2005). Keys to the families of Cicadomorpha and subfamilies and tribes of Cicadellidae (Hemiptera: Auchenorrhyncha). Florida Entomologist.

[B7385939] Dlabola J (1974). Ergebnisse der Tschechoslowakisch-Iranischen Entomologischen Expedition nach dem Iran 1970. Acta Entomologica Musei Nationalis Pragae.

[B7297120] Dlabola J (1981). Ergebnisse der Tschechoslowakisch-Iranischen Entomologischen Expeditionen nach dem Iran (1970 und 1973). Acta Entomologica Musei Nationalis Pragae.

[B7381302] Fieber F X (1969). Synopse der Europäischen Deltocephali. Bull. Verhandlungen der Zoologisch-Botanischen Gesellschaft in Wien.

[B7381188] Knight W J (1965). Techniques for use in the identification of leafhoppers (Homoptera: Cicadellidae). Entomologist’s Gazette.

[B7297138] Linnavuori R (1965). Studies on the South and East mediterranean Hemipterous fauna. Acta Entomologica Fennica.

[B7379627] Metcalf Z. P. (1967). General catalogue of the Homoptera. Fascicle VI. Cicadelloidea..

[B7297186] Nast J (1972). Palaearctic Auchenorrhhyncha (Homoptera) an annotated checklist.

[B7315084] Nielson MW, Maramorosch K, Harris KF (1979). Leafhopper vectors and plant disease agents.

[B7381179] Oman P W (1949). The Nearctic leafhoppers (Homoptera: Cicadellidae). A generic classification and check list. Memoirs of the Washington Entomological Society.

[B7383110] Ossiannilsson F (1983). The Auchenorrhyncha (Homoptera) of Fennoscandia and Denmark. Part 3: the family Cicadellidae: Deltocephalinae, catalogue, literature and index. Fauna Entomoligica Scandinavica.

[B7297147] Puton A (1877). Descriptions de deux nouvelles espèces d'Hémiptères. Bulletin de la Société Entomologique de France.

[B7297178] Ribaut H (1952). Homopteres Auchenorhynques, II (Jassidae), Faune de France.

[B7297056] Weintraub PG, Beanland L (2006). Insect vectors of phytoplasmas. Annual Review of Entomology.

[B7381210] Zahniser J N An online interactive key and searchable database of Deltocephalinae (Hemiptera: Cicadellidae). http://zahniser.speciesfile.org/.

[B7297000] Zahniser J N, Dietrich C H (2008). Phylogeny of the leafhopper subfamily Deltocephalinae (Insecta: Auchenorrhyncha: Cicadellidae) and related subfamilies based on morphology. Systematics and Biodiversity.

